# Production and Status of Bacterial Cellulose in Biomedical Engineering

**DOI:** 10.3390/nano7090257

**Published:** 2017-09-04

**Authors:** Mona Moniri, Amin Boroumand Moghaddam, Susan Azizi, Raha Abdul Rahim, Arbakariya Bin Ariff, Wan Zuhainis Saad, Mohammad Navaderi, Rosfarizan Mohamad

**Affiliations:** 1 Department of Bioprocess Technology, Faculty of Biotechnology and Biomolecular Sciences, Universiti Putra Malaysia, 43400 UPM Serdang, Selangor, Malaysia; mona_moniri6@yahoo.com (M.M.); amin.broomandm@yahoo.com (A.B.M.); arbarif@upm.edu.my (A.B.A.); 2 Young Researcher and Elite Club, Sabzevar Branch, Islamic Azad University, Sabzevar, Iran; 3 Department of Cell and Molecular Biology, Faculty of Biotechnology and Biomolecular Sciences, Universiti Putra Malaysia, 43400 UPM Serdang, Selangor, Malaysia; raha@upm.edu.my; 4 Bioprocessing and Biomanufacturing Research Center, Faculty of Biotechnology and Biomolecular Sciences, Universiti Putra Malaysia, 43400 UPM Serdang, Selangor, Malaysia; 5 Department of Microbiology, Faculty of Biotechnology and Biomolecular Sciences, Universiti Putra Malaysia, 43400 UPM Serdang, Selangor, Malaysia; zuhainis@upm.edu.my; 6 Institute of Tropical Forestry and Forest Products, Univerciti Putra Malaysia, 43400 UPM Serdang, Selangor, Malaysia; 7 Young Research and Elite Club, Parand Branch, Islamic Azad University, Parand, Iran; navaderimohammad@yahoo.com

**Keywords:** bacteria cellulose, bacteria nanocellulose, wound dressing, scaffolds, biosensors

## Abstract

Bacterial cellulose (BC) is a highly pure and crystalline material generated by aerobic bacteria, which has received significant interest due to its unique physiochemical characteristics in comparison with plant cellulose. BC, alone or in combination with different components (e.g., biopolymers and nanoparticles), can be used for a wide range of applications, such as medical products, electrical instruments, and food ingredients. In recent years, biomedical devices have gained important attention due to the increase in medical engineering products for wound care, regeneration of organs, diagnosis of diseases, and drug transportation. Bacterial cellulose has potential applications across several medical sectors and permits the development of innovative materials. This paper reviews the progress of related research, including overall information about bacterial cellulose, production by microorganisms, mechanisms as well as BC cultivation and its nanocomposites. The latest use of BC in the biomedical field is thoroughly discussed with its applications in both a pure and composite form. This paper concludes the further investigations of BC in the future that are required to make it marketable in vital biomaterials.

## 1. Introduction

**Figure 1:**
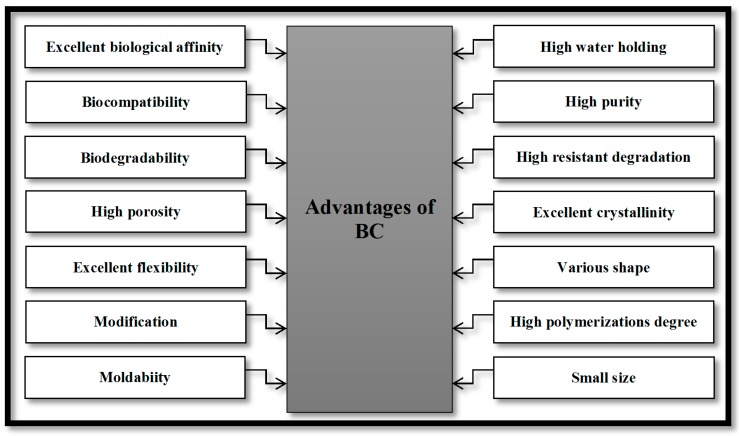
Advantages of bacterial cellulose.

Cellulose is one of the most plentiful biopolymers on earth, representing about 1.5 trillion tons of the total annual biomass production. Cellulose exists in cotton, wood, hemp, and other plant-based materials, and serves as the dominant reinforcing phase in plant structures. Cellulose is also synthesized by algae, tunicates, and some bacteria [1,2,3]. Bacterial cellulose (BC) is a purified form of cellulose, which is produced by several types of bacterial species that mainly belong to the genus *Acetobacter* [4,5]. BC consists of microfibrils, which are free of lignin and hemicellulose. These microfibrils are arranged in a 3D web-shaped structure, providing a porous geometry and high mechanical strength [6,7]. Compared to plant cellulose, BC has considerably higher crystallinity (80–90%) [5], water absorption capacity [8], and degree of polymerization (up to 8000) [9] (Figure 1). These characteristic properties, along with its biocompatibility, make it an attractive candidate for a broad range of applications in various fields, particularly those associated with biomedical and biotechnology applications [9].

Although BC is a biomaterial of prime importance in many fields, pure BC possesses certain restrictions that limit its application, including in the medical and industrial field [7,10,11,12]. The limitations related to the pure polymers can be effectively overcome by the use of composites. BC composites showed considerably improved properties, leading to additional applications in the medical and other industrial fields. In this issue, numerous efforts have been made to produce BC composites using various materials as well as to promote its properties and applications [6,10,13].

Currently, studies are focused on the aspects of practical applications. The materials containing composites are generally synthesized for targeted applications. To this end, BC composites have exhibited notable results, particularly in biomedical fields. BC and its composites have been applied in several medical applications, such as wound healing, skin and tissue regeneration, healing under infectious environments, development of artificial organs, blood vessels, and skin substitutes [10,14,15].

This review includes a summary of the recent advancements reported in BC production as well as a snapshot of the research on BC in order to apply BC and its composites in the medical field. First, the cellulose resources are introduced. Before the process, methods to obtain the BC and the corresponding morphologies are described. The BC cellulose composites are introduced. Finally, its practical and potential applications for medical application of BC composites in wound care, tissue engineering, diagnostic biosensors, and drug delivery are discussed.

## 2. Bioproduction of Bacterial Cellulose

### 2.1. Type of Bacteria

BC is a highly crystalline linear biopolymer of glucose, which is produced with a width of less than 100 nm primarily by the bacterium *Gluconacetobacter xylinus* (*G. xylinus*) (formerly named *Acetobacter xylinus*) in both synthetic and non-synthetic mediums through oxidative fermentation [16,17,18,19]. This non-photosynthetic organism can obtain sugar, glycerol, glucose, and some other organic materials, before changing them into pure cellulose [20,21]. Very few species of bacteria can produce cellulose. However, the gram-negative bacterium *G. xylinus* secretes a large quantity of cellulose as microfibrils from a row of synthetic sites along the longitudinal axis of the cell [22,23]. This aerobic gram-negative bacterium is efficiently fermented at a pH of 3–7 and in a temperature range of 25–30 °C, using saccharides as a carbon source [24]. The superior physicochemical properties of BC are mainly the result of its microfibrillar structure, such as excellent tensile strength, high degree of polymerization, and crystallinity. The microfibrils from each synthetic site assemble to form a large ribbon of cellulose in the growth medium. The formed ribbons and the associated cells intertwine to form a floating pellicle, which allows this stable and strictly aerobic bacteria to develop in the higher oxygen pressure found at the surface of the growth medium [25,26]. Although production of BC has been mainly studied in *G. xylinus*, some microorganisms, such as other species of *Agrobacterium tumefaciens*, *Gluconacetobacter*, *Rhizobium* spp., and gram-positive *Sarcina ventriculli*, also show the ability to produce this biopolymer [27,28].

#### 2.1.1. *Agrobacterium tumefaciens*

The plant pathogen *Agrobacterium tumefaciens* (*A. tumefaciens*) is a free-living soil organism that is readily isolated and grown in the laboratory. Glucose metabolism by this gram-negative, obligate aerobe involves the Entner–Doudoroff pathway (55%) and the pentose cycle (44%) [29]. Cultures of this organism synthesize cellulose from a wide diversity of substrates. Cellulose synthesis in *A. tumefaciens* is apparently a constitutive feature of the genus, because cells synthesize cellulose during growth in the absence of plant cells [30,31] and resting cells maintain a high capacity for cellulose synthesis [30]. That cellulose synthesis is an intrinsic property of *A. tumefaciens*, which is demonstrable in the ability of resting cells to produce cellulose and on a genetic basis.

#### 2.1.2. *Rhizobium* spp.

The roots of leguminous plants are associated with bacterial nodules in possibly one of the most important symbiotic relationships of the biosphere [32]. The atmospheric nitrogen-fixing rhizobia occur as both free-living and colonizing bacteria, which generally display a high degree of specificity for their plant host. The tremendous amounts of energy required for nitrogen fixation are manifested in the catabolic sequences of this aerobic, gram-negative bacterium, Although the fast-growing strains are capable of growth on a wide variety of carbon substrates, the more limited, slow-growing strains exhibit great nutritional diversity with respect to aromatic substrates [33]. While all strains use the Entner–Doudoroff pathway and Krebs cycle, the pentose phosphate pathway is restricted to the fast-growing strains [33].

Cellulose synthesis is an intrinsic property of *Rhizobium* strains. Cells produce cellulose fibrils as an extracellular product during growth in the absence of plant cells. Fibril formation is strongly dependent on the growth phase [34,35]. The cellulose fibrils produced by *Rhizobium leguminosarum* (1 to 10 per cell) range from 5 to 6 nm in diameter and up to 10 µm in length [34]. During cultivation in standing cultures, a surface pellicle is formed [34].

#### 2.1.3. *Sarcina ventriculi*

*Sarcina ventriculi* is a gram-positive, obligate anaerobic soil bacterium, which is capable of growing on sugars within an extraordinarily wide pH range (from 2 to 10) [36]. The cellulose produced by *S. ventriculi* accumulates outside the cell. The cellulose of *S. ventriculi* remains closely associated with the cell wall and has the structural function of tightly binding the cells into large packets. Cellulose is not deposited in the cell walls of *S. ventriculi*. Removal of cellulose from the packets with Cross and Bevan’s reagent does not affect the cell walls. Cells from which the cementing material is removed still possess intact cell walls but no longer take up the cellulose stain [37]. In the small aggregates of cells obtained when large packets are treated with Cross and Bevan’s reagent, the remaining cellulose is most likely tightly constricted by the adjacent cells, resulting in the solvent not being able to effectively act upon it.

### 2.2. Mechanism of Cellulose Synthesis and Purification

*Acetobacter xylinum* (*A. xylinum*) has been extensively used as a model for the fundamental and applied investigations on cellulose due to its capability to synthesize relatively high numbers of polymers from a widespread range of carbon and nitrogen resources. 

Cellulose-producing bacteria function in the pentose-phosphate cycle or the Krebs cycle, depending on the physiological state of the cell coupled with gluconeogenesis [22]. The pentose–phosphate cycle involves the oxidation of carbohydrates, while the Krebs cycle involves the oxidation of acetate-derived carbohydrates, fat and proteins, such as oxalosuccinate and a-ketoglutarate. However, *A. xylinum* is not able to metabolize glucose anaerobically because it lacks phosphofructose kinase, which is required for glycolysis [38]. Numerous authors have reported the biosynthesis of cellulose by *A. xylinum* [39,40,41,42,43,44]. The mechanism of transforming the glucose to cellulose through *A. xylinum* involves biosynthetic processes of cellulose. Synthesis of bacterial cellulose is an exact and specific regulated multi-step route that includes a large amount of both singular enzymes as well as sets of catalytic and regulatory proteins. However, its supramolecular structure has not yet been well recognized [45].

The biosynthesis of BC requires four enzymatic steps: (1) phosphorylation of glucose by glucokinase to glucose-6-phosphate; (2) isomerization of glucose-6-phosphate to glucose-1-phosphate by phosphoglucomutase; (3) conversion of glucose-1-phosphate to uridine diphosphate glucose (UDP-glucose) by UDP-glucose pyrophosphorylase; (4) and the synthesis of cellulose from UDP-glucose by cellulose synthase [22]. UDP-glucose is the direct cellulose precursor, which is common in many organisms [46]. UGPase is thought to play an important role in cellulose synthesis since it is approximately 100 times more active in cellulose producers than that of non-cellulose producing bacteria [47]. When disaccharides, such as sucrose and maltose, are used as a carbon source for cellulose-producing bacteria, the biosynthesis of BC starts with the hydrolysis of disaccharides into monosaccharides, such as glucose and fructose. Although the pathways and mechanisms of UDPGlc synthesis are relatively well known, the molecular mechanisms of glucose polymerization creating long and unbranched cellulose chains are still elusive to scientists [47]. 

**Figure 2:**
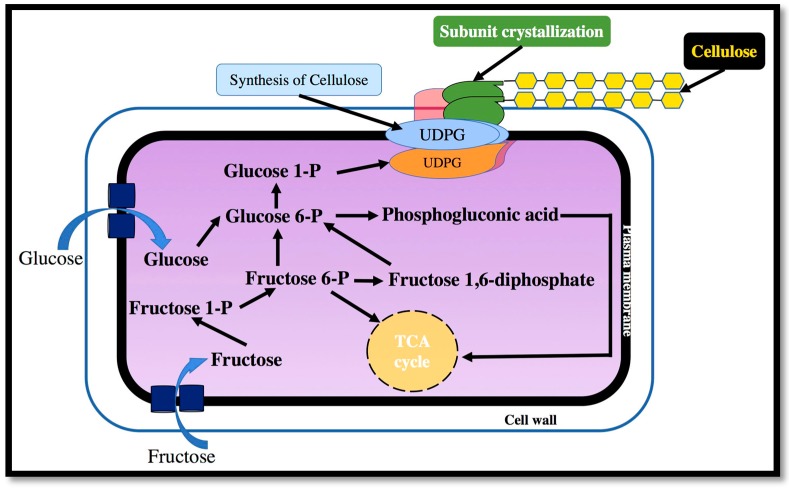
Biosynthetic pathway of cellulose in the cells of microorganisms.

The synthesis of cellulose in microorganisms and plants follows two intermediate stages: (i) creation of β-1,4-glucan chain by polymerization of glucose units; and (ii) synthesis and crystallization of the cellulose chain [15]. The general mechanism for the cellulose biosynthetic pathway is shown in Figure 2. BC is formed between the outer and cytoplasm membranes of the cell [48]. The cellulose molecules are first synthesized inside the bacteria. These molecules are then spun through cellulose exporting components to form protofibrils, which are approximately 2–4 nm in diameter. A ribbon shaped microfibril of approximately 80 nm is assembled from these protofibrils [49]. 

The biosynthesis of cellulose is catalyzed by cellulose synthase, which polymerizes the glucose units into the 1,4-β-glucan chains. However, the polymerization mechanism of converting glucose monomers to glucan chains is not yet well understood. Two hypotheses for this mechanism in *G. xylinus* have been reported. The first hypothesis assumes that the polymerization of β-1,4 glucan involves a lipid intermediate. The involvement of the lipid intermediate in the synthesis of acetan, a soluble polysaccharide, has been proven [50]. Studies have to be carried out to determine whether the lipid intermediate plays any role in cellulose synthesis. The polymer synthesis is catalyzed by purified cellulose synthase in *A. xylinum* subunits, which does not contain the lipid component [51]. Another hypothesis was reported by Brown and Saxena [52], that the polymerization of the β-1,4 glucan does not involve a lipid intermediate. The glucose residues were added to the non-reducing end of the polysaccharide, with those reducing ends being nascent polymer chains that were situated away from the cells.

**Figure 3:**
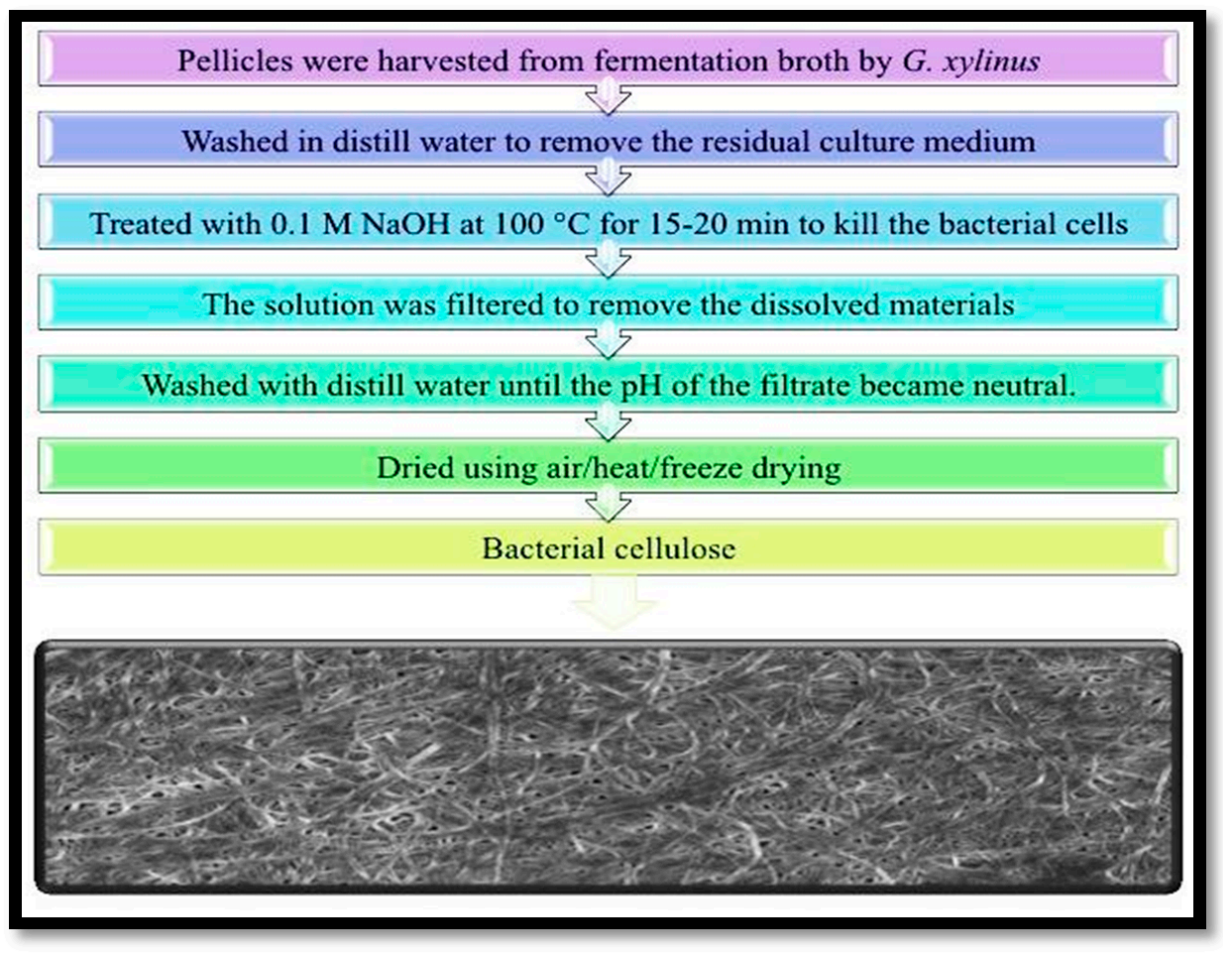
The process of purification of bacteria cellulose (BC) from *G. xylinus*.

After fermentation, the obtained microbial cellulose is not pure and thus, requires purification. This is the result of some impurities, such as cells and/or the medium constituents. The fermented medium is required to be purified to acquire pure cellulose. The procedure of purification of BC is defined in Figure 3 [53]. The most extensive applied process of purification of BC in the culture medium involves using alkaline substances, such as sodium hydroxide and potassium hydroxide, and organic acids, such as acetic acid; or washing the mixtures several times with reverse osmosis water or hot water. The above cited purification stages can be applied separately or together [54]. A typical purification procedure of BC containing captured cells includes the following steps: (i) treatment with solutions, such as NaOH/KOH/Na_2_CO_3_, at 100 °C for 15–20 min to remove the microbial cells; (ii) filtration of solution using an aspirator to eliminate the dissolved materials; and (iii) frequently washing of the filtrate with distilled water until its pH becomes neutral.

### 2.3. Methods

There are two main approaches to synthesize BC using microorganisms: (i) static culture, which results in the growth of a dense, coriaceous white BC pellicle at the air–liquid interface [55,56]; and (ii) stirred culture, in which cellulose is produced in a dispersed procedure in the culture medium, creating anomalous pellets or suspended microfibers [57,58,59]. The selection of the method (i.e., static or stirred culture) depends on the final application of BC as the physical, morphological and mechanical characteristics of the synthesized BC vary based on the cultivation process. For instance, the obtained cellulose via the stirred culturing method possesses a lower mechanical strength compared to the cellulose produced by static culture. Furthermore, the stirred culturing method results in lower yields in comparison with the static culture and a higher mutation in the microorganism is probable, which can affect the production of BC [56,60,61]. On the other hand, the static culture needs a larger cultivation area and a longer culturing period [62,63,64,65,66].

### 2.4. Medium 

The most important factor to determine the final price of BC is the culture medium. Thus, one significant aspect in BC synthesis is finding a low-priced culture medium, which can enhance the yield of BC and be applied as an economically feasible solution for use in a wide range of fields [67]. Recently, studies have mostly focused on enhancing the utilization of industrial and agricultural wastes as a substitution for nutrient sources in order to make this production both “greener” and low-cost. The bioreactor design and medium agitation are important factors [68]. There are three different types of media, including the Hestrin–Schramm (H) [69], Yamanaka (Y) [70], and Zhou (Z) [13] media. The optimized concentrations have been formerly reported for these media. The Hestrin–Schramm medium is an important and effective method, which must be supported in order to limit the use of high-cost raw materials in the fermentation process and substitution of them with some cheap materials. Useful materials could include industrial, agricultural and forestry by-products and waste materials, which are counted as low-cost non-conventional sources of carbon and/or nitrogen [24,71,72,73,74]. The final dry weight of the obtained BCs in the Zhou (Z) and Hestrin–Schramm (H) media is higher than that of BC synthesized in the Y medium [60]. Although the carbon source of H medium is half that of the Z medium, it produces the highest yield of BC. Thus, it can be concluded that the yield of BC production is affected by type, quantity and composition of the medium. The production of BC by microorganisms, such as *G. xylinum*, is influenced by a variety of important parameters, such as the applied culture method, the source of carbohydrates, the strain of bacteria, acidity, and temperature [75]. In comparison with the other two media, the pH in the H medium declined the least. Thus, the H medium is more buffered than the other two media. This outcome explains the high BC yield production in the H medium. The formation of gluconic acid as a byproduct of the medium containing glucose is the reason for the reduction in pH [62]. It was reported that the BC fibrils grown in Z medium were more tangled than those formed in H and Y media. It was also found that the width of BC fibrils created in H medium was broader than those of the formed fibrils in the Y and Z media. Another finding indicates that the crystallinity of BC may be controlled by the type of applied medium and culturing conditions [60]. The BC produced in H and Y media showed the maximum and the minimum crystallinity, respectively. Similar findings were obtained for the glucose consumption.

Recent studies have mostly focused on the relationship between BC characteristics and carbon source, which is important for its applications [76,77,78]. The findings have mainly showed that although the chemical structure of the produced BC is not changed, the used carbon source can have an effect on the water holding capacity (WHC) of the membranes (WHC has a positive and significant correlation with the BC surface area and porosity), their level of polymerization, molecular weight, crystallinity index (67–96%), mean crystallite size (5.7–6.4 nm), intrinsic viscosity, oxygen and water vapor transmission rates, as well as mechanical properties [74,79,80,81,82,83]. Several studies reported that high quantities of cellulose were synthesized when glucose, fructose and mannitol were utilized as carbon sources. According to the above-mentioned findings, for a selected culture composition, the duration of incubation time has a significant effect on the production and characteristics of the produced BC, such as membrane porosity, pellicle compactness, crystallinity, and mean degree of polymerization [84,85].

### 2.5. Morphology

It was established that the cellulose were fabricated by elementary fibrils, namely microfibrils and macrofibrils classified by the morphological view [86]. There are two main regions in cellulose: high- and low-ordered regions. Microfibrils of cellulose are fabricated by the high-ordered region, which is crystalline. However, the low ordered macrofibrils are composed of crystalline and non-crystalline districts. 

One approach to change the morphology of BC involves the modification of bacterial cells. A useful procedure to match BC characteristics with its applications involves the manipulation of BC biogenesis. This approach has been followed in a few studies. For example, modified BC was produced by growing BC cells in a chitosan-modified growth medium for the application of wound dressing in a previous study [87]. In another study, Seifert et al. [88] made modified BC in a methylcellulose, carboxymethyl cellulose, and poly(vinyl alcohol)-modified medium to fabricate BC with controlled water content for medical applications.

Nano-sized BC composites can be produced by biogenesis manipulation, which aims to allow the BC fibrils and polymer to co-crystallize throughout the ribbon formation process. Bodin et al. produced BC tubes with various morphologies based on the product requirements, which made it an interesting biomaterial to be explored for utilization in different biomedical applications, including bone graft material as well as scaffolds for tissue engineering of cartilage and blood vessels [89]. Beyond manipulating some of the morphological characteristics of BC, such as shape and size, the microscopic morphology may also be altered in some ways. Watanabe and Yamanaka [90] and Hult et al. [91] have showed that a high oxygen ratio during static cultivation can increase the density and consequently, the toughness of the BC membrane. Quivering velocities and adding dyes have also showed a profound effect on the combination of the sub-elementary microfibrils, which subsequently reduces the crystallinity of the BC network [61,92].

Different morphologies of BC have a direct effect on its mechanical properties and cell attachment into the material. Tang et al. [93] produced BC mats with different aperture sizes and porosities when the fermentation conditions (i.e., cultivation time and inoculation volume) and post-treatment interventions (alkali treatment and drying methods) were changed. Backdahl et al. [94] invented a novel approach to introduce microporosity in BC tubes, which are intended to be used as scaffolds for tissue-engineered blood vessels. In the approach, paraffin wax and various sizes of starch particles were placed in a culture medium of *Acetobacter xylinum* to prepare scaffolds made of bacterial cellulose that had various morphologies. Grande et al. [95] introduced self-assembled nanocomposites of cellulose, which were produced by the *Acetobacter* bacteria and starch.

## 3. Bacterial Cellulose Nanocomposites

**Figure 4:**
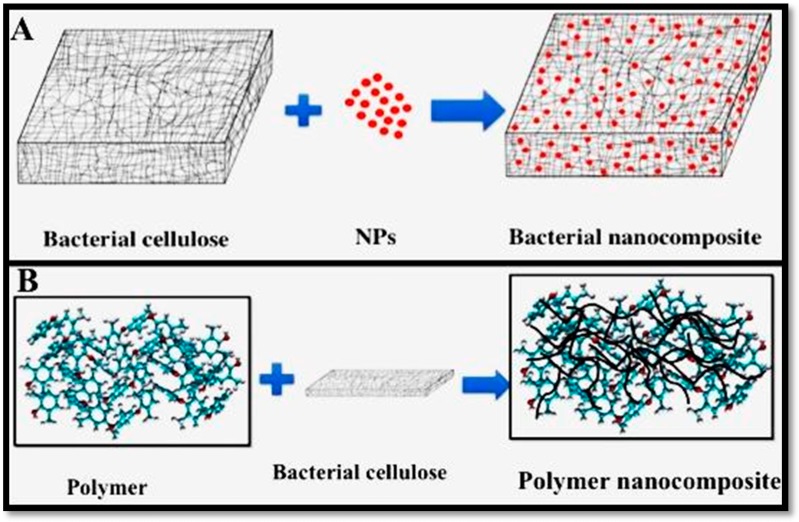
The use of BC as a (**A**) matrix or (**B**) reinforcement to prepare nanocomposites.

Recently, nanocomposites have received remarkable attention and have been widely studied due to their excellent properties. As a significant type of nanocomposite, cellulose-based nanocomposites have also attracted much interest. Typically, composites consist of two types of individual materials, which are namely the matrix and the reinforcement material [10,96,97,98]. The matrix acts as a scaffold and supports the reinforcement material, while reinforcements impart the physico-chemical and biological properties to the matrix. Bacterial cellulose can be used as both the matrix and scaffold (Figure 4) for the composite design. 

Bacterial cellulose is a suitable substrate to house a range of nanomaterials due to its highly porous structure, high specific surface area and mechanical strength. Some BC composites have been prepared with the aim to overcome the deficiencies and improve the biological, physiological, mechanical and chemical properties of BC [4,8,96,98,99,100,101,102,103]. It has been proven that the amalgamation of nanomaterials (Au, Ag, ZnO, etc.) and polymers (PAni, chitosan, PEG, etc.) can help to synthesize BC composites with bactericidal and conductive properties [10]. Generally, three different approaches have been used to incorporate nanomaterials into/onto the BC matrix: (1) direct addition of nanomaterials into a BC matrix; (2) synthesis of nanomaterial in the structure of BC-based materials; and (3) direct coating of nanomaterials as a nano-layer on the BC surface. Until now, many nanomaterials, such as metal and metal oxides nanoparticles (Ag, Au, Ni, Pd, CuO and TiO_2_), mineral nanomaterials (SiO_2_, CaCO_3_, montmorillonite) and carbonaceous nanomaterials (grapheme, carbon nanotube) have been housed into nanocellulose matrices for preparing BC nanocomposites (Table 1). 

In recent years, it has been reported that highly crystalline BC has a unique and outstanding potential for increasing the composite material properties at lower filler concentration in comparison to the unfilled polymer matrix and to their microcomposite counterparts [104,105].

There are numerous papers in the literature that involve the production of cellulose composite materials. Hutchens et al. published a patent involving the use of composite bacterial cellulose as biomaterials. They incubated bacterial cellulose in a solution of sodium hydrogen phosphate and calcium chloride in latex. The X-ray diffraction confirmed the incorporation of calcium and phosphate in the bacterial cellulose. Cellulose has been utilized as both reinforcing material and matrix [106]. The cleanly arranged fibrous network structure of cellulose can cage in NPs and thus, it acts as a matrix [100,101]. Similarly, cellulose nanofibers can reinforce other polymeric and non-polymeric materials, such as cells and tissues [107,108]. Bacterial cellulose has shown very promising characteristics for its use as reinforcement material for composites with optical functions. Recently, a study showed that the characteristics of the composite formed, such as transparency and low coefficient of expansion, are due primarily to networks of chains of semicrystalline materials nanofibers produced by the bacterium *Acetobacter xylinum* [109]. Ifuku et al. obtained acetylated BC sheets to enhance the properties of optically transparent composites of acrylic resin reinforced with the nanofibers [106]. BC has similar chemistry and superior physical properties to plant cellulose and has therefore been used in the preparation of a number of composite materials for various applications. 

## 4. Bacterial Cellulose in Biomedical Applications

**Figure 5:**
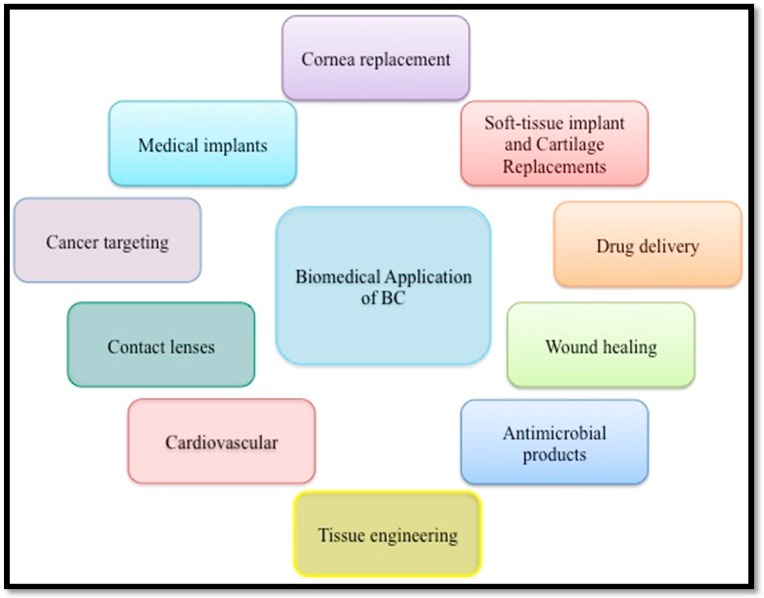
Biomedical applications of BC.

The ideal structure, biocompatibility, and sustainability of BC have led to many studies and prompted its application in a variety of fields, such as medical, food, paper, textiles, and electronics [5,7,99,134]. Nowadays, BC-based materials are mostly used in the medical field, including in wound healing materials, artificial skin and blood vessels, scaffolds for tissue engineering, and drug delivery (Figure 5) [14,15,19,135,136].

### 4.1. BC-Based Materials for Wound Healing 

One of the well-known clinical applications of BC is wound dressing. Burns are very dangerous injuries that cause extensive destruction of skin tissue. Some factors, such as cellular and biochemical ingredients as well as enzymatic pathways, play essential roles during the recovery stage of the wounded tissue. Normally, a typical wound healing process encompasses a series of steps, including homeostasis, inflammation, granulation tissue growth (proliferation), and remodeling (maturation) [137]. According to the findings of several studies, BC has been introduced as an excellent biomaterial in conventional wound dressing. There are some BC-based dressing materials, such as Bioprocess, XCell, and Biofill, which are available in the market commercially [138]. 

The findings of several studies show that the topical application of BC-based wound dressing enhances the healing process of burns and chronic injuries. In 1962, George Winter found that healing and particularly re-epithelization was accelerated when a wound was maintained in a moist environment [139]. Since then, efficient wound dressings are mostly designed to maintain moisture on the affected part. A higher permeability of moist dressings to water is an advantage for wound healing. In a study, Czaja et al. treated patients, who suffered from severe second-degree burns with BC membranes [15]. It was reported that the skin of patients whose wounds were treated with a BC membrane improved faster than those patients who used conventional wound dressings. Czaja et al. found that non-dried BCs exhibited notable compatibility for different body types, maintained a suitable water balance, and considerably reduced wound pain. 

More recently, animal studies by Fu et al. also confirmed the effects of BC-based wound dressing materials in accelerating tissue regeneration and healing in addition to reducing the inflammatory response [140]. Moreover, outcomes of several studies show that the topical use of microbial cellulose (MC) membranes accelerates the process of wound healing. Another study also reported that MC membranes showed better efficiency compared to conventional wound dressings in: (1) compatibility with the wound surface (excellent covering to all facial contours and a high level of cohesion even to the contoured regions, such as mouth and nose, were seen); (2) preserving a moist environment in the wound; (3) significant reduction in pain; (4) accelerating re-epithelialization and the generation of granulation tissue; and (5) reduction of scar formation [136,141].

Until now, gauze dressing has been extensively used in clinical wound dressings. Recently, Fu et al. [142] has used BC as a potential skin-tissue healing material in vivo to substitute traditional gauze dressings [142]. Pathological investigations showed that there were better and accelerated healing influences as well as a reduced inflammatory response in thick BC compared to thin BC. Histological studies showed remarkable capillary formation, tissue regeneration and cell proliferation in the wound region in the thick-BC materials [142]. In a recent study, BC wound-dressings have been compared to two types of commercial dressings, namely Algisite M and Vaseline gauze, in a rat model [143]. This study revealed that the BC-dressed animals had more prompt wound healing without any toxicity in comparison with other groups. This established the efficiency of BC-based dressing materials for clinical applications. 

#### BC-Based Wound Dressing with Antimicrobial Efficiency

An important reason for prolonged wound healing is infection, which is caused by high bacterial levels. Adherence and growth of pathogenic bacteria on the wounds lead to concomitant contagion to new hosts. This considerably facilitates the proliferation of bacteria, which poses a significant threat to human health. Considering the increasing importance of infectious illnesses and antibiotic resistance, many investigations have been dedicated to developing efficient surface disinfection. Thus, new wound dressing materials with antimicrobial and other bioactive properties have been produced.

**Scheme 1:**
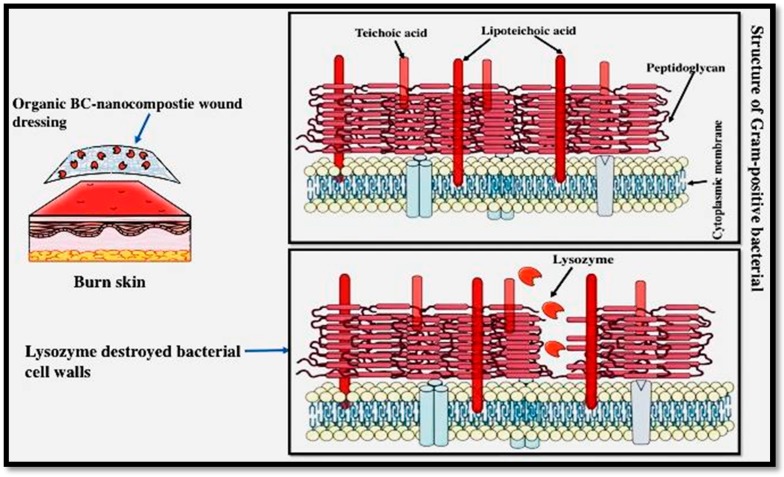
Organic nanocomposites for wound healing.

Nanocellulose with a porous network structure in the architecture of biomaterials is suitable for delivering antibiotics and other medicines to the wound in addition to serving as an effective barrier against any external infection [144]. It has also been found that antimicrobial nanomaterials from nanocellulose (carbohydrate nature) generally show compatibility with biological tissues as well as satisfactory bioavailability and biodegradability [111,145]. However, due to BC-based materials having no antimicrobial activities, BC-based materials cannot inhibit wound infections. Thus, BC-based antimicrobial composites are generally produced by the combination of antimicrobial agents and BC using physical or chemical methods. Based on various types of antimicrobial agents, BC-based antimicrobial materials can be categorized into two groups: BC incorporated with inorganic materials (i.e., Ag, ZnO, CuO particles, and their derivatives) and organic antimicrobial agents (e.g., lysozyme). Enzymatic degradation assessments revealed that modified BC is susceptible to lysozyme decomposition (Scheme 1). Moreover, modified BC has bacteriostatic ability against gram-positive pathogens. 

Jebali et al. [146] investigated the antimicrobial ability of nanocellulose combined with lysozyme and allicin. Pure nanocellulose showed lower antibacterial activity when compared to the nanocellulose containing lysozyme and allicin. These two sets of materials, namely the allicin-conjugated nanocellulose and lysozyme-conjugated nanocellulose, showed excellent antimicrobial properties against the standard bacterial strains of *Staphylococcus aureus* and *Escherichia coli* as well as the fungal strains of *Aspergillus niger* and *Candida albicans*.

**Scheme 2:**
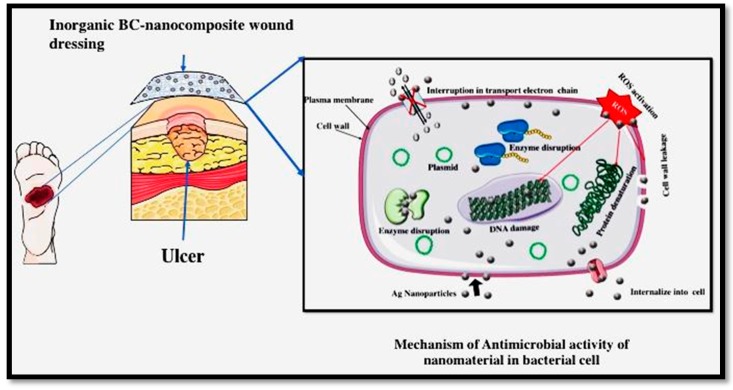
BC impregnated with Ag nanoparticles for the wound dressing and interaction of Ag nanoparticles with bacteria cell pathogens.

Amongst inorganic materials, silver has been most widely probed and utilized against infections since ancient times. There are several pathways in which silver ions kill bacteria: (i) interaction of silver ions with the thiol groups of enzyme and proteins, which are significant for bacterial respiration and transportation of essential substance through the cell wall and inside the cell [147,148]; and (ii) attachment of silver ions to the bacterial cell membrane and external bacterial cell, changing the function of the bacterial cell wall [149]. Hence, silver metal and its compounds are effective materials in preventing bacterial infections [150]. Silver metal is gently changed into silver ions under the physiological system and interacts with bacterial cells. It has been proven that silver nanoparticles with effective antibacterial, antifungal and antiviral characteristics are attractive antibacterial agents [151] (Scheme 2). It was found that the antimicrobial efficiency of Ag nanoparticle in BC-based materials is affected by the size and shape of the applied nanoparticles. 

In a study, Maneerung et al. [111] used silver nanoparticles to create an antimicrobial activity in the BC dressing for preventing wound infection. For this purpose, BC was soaked in a silver nitrate solution, before the absorbed silver ions (Ag^+^) within the BC network were reduced to metallic silver nanoparticles (Ag^0^) by sodium borohydride. Findings indicated that the freeze-dried silver nanoparticle-impregnated BC revealed significant antimicrobial affect against both gram-negative (*Escherichia coli*) and -positive (*Staphylococcus aurous*) bacteria.

### 4.2. BC-Based Composite for Tissue Engineering 

Another area of innovation and potential exciting application for BC material is tissue engineering [152]. Tissue engineering requires an intertwined interaction between the scaffold materials, cells and surroundings in order to generate a new tissue. Due to the unique characteristics of BC, including the 3D network structure, potential biocompatibility and significantly low cytotoxicity, excellent mechanical properties as well as highly porous structure, BC is the most suitable material for tissue engineering applications [108,153,154]. Therefore, BC has mostly been used as a scaffold in tissue engineering applications. Petersen and Gatenholm have explained that the biocompatibility of BC can be ascribed to the BC structure, which is similar to extracellular matrix components, such as collagen. In fact, BC and collagen have comparable diameters (about 100 nm). Furthermore, they are both formed extracellularly from precursor monomers into polymer chains. The mechanical properties of BC gels with collagen meniscal implants and real pig menisci obtained from pigs were compared in a study. It was found that the mechanical properties of the both BC gels were relatively equal [155].

One problem in using polymer-based scaffolds in bone tissue engineering is the inability to preserve their high-strength stability. To solve this issue, chemical crosslinking is mostly used to stabilize these scaffolds. Thus, it is a challenge to produce load-bearing scaffolds for bone tissue engineering applications. The main advantages of cellulose for bone tissue engineering are its biocompatibility and high mechanical properties. BC and its composites have been studied as an alternative for the cartilage due to their similar properties [138]. In a study, PVA containing BC nanofibers (<1%) nanocomposites were prepared and exposed to thermal cycling [156]. The fabricated PVA–BC nanocomposites were investigated as potentially improved substitution materials for articular cartilage. The PVA–BC nanocomposites exhibited tunable compressive mechanical characteristics with elastic modulus magnitudes closer to that of the original articular cartilage. The nanocomposites similarly exhibited enhanced strain rate dependence and suitable viscoelastic properties. The PVA–BC nanocomposites displayed attractive properties for replacing localized articular cartilage injuries and many other orthopedic problems, such as damaged intervertebral discs. Based on the high mechanical properties, compatibility, foldability, and low price, the BC-based artificial endocranium (AE) was prepared and subsequently patented [157]. In this study, the BC wet sheet was harvested from fermentation of *G. xylinus*, before being crushed into powder form. After that, PVA, sodium alginate and carboxy methyl cellulose (CMC) were mixed with a homogenous BC suspension to obtain a moldable liquid. The mixture was then molded and freeze-dried to fabricate the AE. The strength at break point of the prepared AE was in the range of 4–5 N/mm^2^, the elongation at the break was from 45% to 98% and the hook strength was from 5 to 8 N/mm^2^.

In a similar study, Kumbar and coworkers [158,159] developed mechanical properties of cellulose scaffold materials for bone tissue engineering applications. The mechanical properties of these scaffolds were in the mid-range of the human trabecular bone and being higher compared to several existing polymer-based bone tissue engineering scaffolds with comparable porous structures under both dry and physiological (wet) situations. These nanocellulose scaffolds with collagen showed improved human osteoblast adherence, proliferation and alkaline phosphatase expression.

#### 4.2.1. BC-Based Composite in Blood Vessel Replacement 

In a normal physiological situation, blood circulates in the vessels throughout the body. However, the vessels become clogged or severely damaged in pathological conditions [160], which should be substituted by other vessels drawn from the patient’s body/donor or artificial ones in some cases. Many researchers have been tried to develop artificial blood vessels to solve these problems [161]. Nevertheless, artificial grafts, such as Dacron (polyester) and ePTFE cause thrombosis. Thus, these grafts are not appropriate alternatives for small blood vessels with a diameter less than 6 mm [20]. BC is a possible material to use for artificial blood vessels for small- or large-sized vascular grafts due to its good mechanical strength (a burst pressure of up to 880 mmHg), porous structure, blood biocompatibility, mold ability and foldability [162]. Pure BC and its composites, such as BC combined with graphene oxide (GO), were formulated to use for this purpose [163]. The GO nanosheets were embedded in the BC nanofibers with hydrogen bonding. The BC–GO composite showed a greater biocompatibility compared to the individually applied substances in addition to inducing cell proliferation. Due to these advantages, BC–GO can potentially be used in artificial blood vessels. In a study, paraffin wax was added to a culture medium of *G. xylinus* to prepare 3D network scaffolds with adjustable microporosity [94]. The BC scaffolds have various morphologies and interconnectivity. In order to obtain microporous BC scaffold, porogens are successfully removed in order to ensure that no residue is detectable in physicochemical analysis. In the future, these microporous scaffolds can be probed for proliferation, differentiation and migration of endothelial cells, which can lead to generation of semi-synthetic or artificial blood vessels. Similarly, Andrade et al. [164] modified BC with chimeric proteins comprised of a cellulose-binding module and adhesion peptides for the synthetic blood vessels. The modified BC was capable to improve endothelial cell adhesion and induce angiogenesis. Leitão et al. [165] showed that BC has an excellent tensile and suture maintenance strength. Therefore, BC can be used for small blood vessels.

Coronary bypass graft surgery is one of the most popular interventions for cardiovascular disease, which is carried out to provide blood to the heart tissue with an appropriate blood vessel substitution. According to the World Health Organization (WHO) and British Heart Foundation (BHF), around 30% of deaths in the world and 42% in the Europe are ascribed to cardiovascular deficiencies and diseases. Annually, several hundred thousand heart bypass surgeries are performed worldwide. Due to the lack of synthetic bypass grafts for this purpose so far, vessels are harvested from the thorax or legs of patients. Usual artificial bypass implants prepared from polytetrafluoroethylene, polyethylene, polyethylene terephthalate, and polyurethane have not been successful for cardiovascular surgery. For the first time, the research group of Dieter Klemm investigated and applied synthetic vascular replacements prepared from BC. They have reported the use of BC as a blood vessel substitute in several publications [1,19,166] and in particular, introduced a medical product called Bacterial Synthesized Cellulose (BASYC) with excellent mechanical strength in the moist state, high water preservation, and smoothness of the internal tube surface. This product has been successfully applied as synthetic blood vessels in animal models for microsurgery [167,168]. Different properties and biology assessment of BC tubes as a blood vessel substitute have been probed, including BC biomaterial-induced coagulation issues [169], cell adhesion, proliferation, viability and invasion [164], hemodynamic analysis, microcirculatory evaluation [170], and so on.

#### 4.2.2. BC-Based Composite in Dental Medicine 

Bacteria cellulose has been examined in dental tissue regeneration. Microbial cellulose prepared by the *G. xylinus* strain may be applied for regeneration of dental tissues in human beings. Nanocellulose products, such as Gengiflex^®^ and Gore-Tex^®^, have intended applications in the dental industry. These products have been made to help periodontal tissue improvement [171]. The Gengiflex^®^ includes two layers: the inner membrane is made of BC, which makes the membrane rigid; while the external alkali–cellulose membrane is chemically modified [172]. A complete improvement in the osseous deficiency around an IMZ implant was reported after Gengiflex^®^ therapy. The advantages, including the restoration of aesthetics and mouth function, reduce a number of surgical steps. Salata et al. [173] used the in vivo non-healing bone-defect model suggested by Dahlin et al. [174] to compare the biological efficiency of Gengiflex^®^ and Gore-Tex^®^ membranes. Findings of this study indicated that Gore-Tex^®^ membranes (a composite of polytetrafluoroethylene, nylon, and urethane) were significantly effective in reducing inflammation, while both membranes accelerated bone growth equally in the same period of time. A significant amount of bone growth was observed for the bone-deficient model by either the Gore-Tex^®^ or BC membrane in comparison to the control groups. However, Gore-Tex^®^ was more tolerated by the tissues compared to Gengiflex^®^. Similarly, Macedo et al. [175] compared BC and polytetrafluoroethylene (PTFE) as physical obstacles used to heal bone deficiencies in tissue regeneration. In the research, two deficient osseous (8 mm in diameter) were made in both posterior feet of four adult rabbits, using surgical blades with continuous irrigation using a sterile saline solution. The created defects on the right posterior feet were protected with PTFE membranes, while Gengiflex^®^ barriers were applied over injuries created in the left posterior feet. After three months, the histological assessment of the interventions showed that the created defects with PTFE membranes were entirely mended with bone tissue. However, incomplete bone growth was observed in the injuries covered with Gengiflex^®^ membranes, resulting in pores and low bone density. According to the results, there is evidence that the non-porous PTFE membrane is a more effective substitute to treat osseous deficiencies than a BC barrier.

### 4.3. Drug Delivery 

Drugs with shorter half-lives must be managed in a controlled manner, which includes several advantages of the dosage form, such as a decrease in dose frequency, relative stability of drug plasma concentration, patients’ satisfaction and therapeutic efficiency [176]. BC and other polymeric materials have been broadly studied for controlled drug delivery. Production of BC-based nanocomposites to optimize the controlled drug delivery is the most important strategy in order to promote the drug-delayed release effects of BC. In some studies, the blend of BC and polyacrylic acid (PAA) (BC–PAA) has been prepared by polymerization initiated through electron beam irradiation using various doses of radiation [177,178]. The degree of swelling in the prepared composites was enhanced with an increase in radiation dose and decrease in ionic strength. In addition, the composites were sensitive to pH and their swelling reached to maximum values at a pH of 7. These BC–PAA composite hydrogels were examined as pH-responsive substances for controlled in vitro drug delivery utilizing various contents of bovine serum albumin (BSA) as a model compound [177]. Moreover, the drug release profiles were controlled using consecutively simulated gastric fluid (SGF) and a simulated intestinal fluid (SIF) without enzymes for 2 h. This is continued until the maximum drug release is obtained. It was seen that the release of drug in SGF was much slower at the end of 2 h. However, the release rate in SIF was significantly higher, but reduced with an increase in the radiation dose. The various releasing rates of SGF and SIF were ascribed to the influence of pH on the swelling rate of the composites. It was found that the pH-responsive behavior of these composites toward drug delivery was similar to the natural BC membranes [177].

The fabricated BC composites were used as a substitute carrier for transdermal drug delivery. The diffusion of drugs through BC membranes was investigated via the diffusion cell technique. The non-irradiated BC membranes showed faster drug diffusion than that of the BC membranes prepared in irradiated conditions. This result was ascribed to the density of pores, which was significantly lesser in non-irradiated samples than those of the irradiated membranes. To evaluate the therapeutic possibility of BC membranes, in vitro penetration analysis of two types of drugs (lidocaine hydrochloride and ibuprofen) was performed through in vitro diffusion studies. The drug release from BC composites was assessed via human epidermis, which was compared with other formulation systems, including ibuprofen gel, aqueous solution, lidocaine hydrochloride gel, and solution in PEG400. Using lidocaine hydrochloride in BC composites resulted in a lower penetration rate compared to those of conventional formulations. In all cases, the water content of the BC membranes was partially lower due to absorption of a solution of the particular drug with glycerol, which causes a plasticizing effect. This makes the BC membrane more flexible and appropriate for transdermal application, rehydration, and swelling of BC [179,180].

In recent studies, many drug delivery systems based on nanocellulose substances for varied pharmaceutical applications have been utilized [181,182,183,184,185,186,187,188,189]. Microbial cellulose has a suitable structure for use in drug delivery systems. Furthermore, BC has a well-supported history of effective use by the U.S. Food and Drug Administration, which is the organization responsible for approving drugs and products. An important purpose of using bacterial cellulose as a suitable exporter of drugs is to manage the rate of drug release and optimize drug concentration.

Mueller et al. [188] studied BC as an effective drug delivery system for proteins with serum albumin. It was found that the freeze-dried BC materials exhibited a lower loading of protein compared to the normal BC ones. Other researchers have investigated the development of BC as a drug delivery system by including drug molecules into the BC material, which permits gradual drug release [190,191,192]. 

### 4.4. Biosensors and Diagnostics

In recent years, biosensors have attracted the attention of researchers in medical field [193]. Thus, many biosensors have been made for application in different fields, including regenerative medicine and tissue engineering [194]. Some factors, such as antibodies, receptors and enzymes, have been broadly utilized in bioanalysis and biosensors as bio-sensing elements [195]. These instruments can control the real-time biological signals in vivo, including the release of antibodies or proteins in response to damaged tissue, inflammatory events, infections, muscular dystrophy or cardiac infarction. Therefore, biosensors have the potential to notify relevant people about the health-care problems in time, which makes them helpful for use in earlier diagnosis of diseases in clinical settings [196]. In a study, the prepared BC-Ag nanocomposites have been used in bioanalysis as Surface Enhanced Raman Scattering (SERS) lamellas using thiosalicylic acid and 2,2-dithiodipyridine as analytes [113]. These nanocomposites were utilized in bioanalysis of l-phenylalanine, l-glutamine, and l-histidine amino acids.

One of the most common biosensors is the blood glucose monitoring device [197]. Moreover, Wang [198] and Wang et al. [199] prepared BC nanofibers containing Au nanoparticles (BC-Au) nanocomposites as a source for amperometric determination of glucose. The fabricated enzymatic glucose biosensor was very sensitive in detecting glucose and was capable of measuring a glucose level as low as 2.3 µM with a linear range of 10–400 µM. This BC–Au-based biosensor was successfully used to measure glucose in human blood. In another study, Wang et al. developed a high efficient biosensor based on nanocellulose–Au nanoparticles hydrogel nanocomposites [200]. Several Heme Proteins (HPs), including hemoglobin, horseradish peroxidase and myoglobin, were fixed on the surface of the nanocomposite. HPs are vital peroxidases, which are composed of iron heme prosthetic groups in their polypeptide pockets. The oxidation of lamella can be catalyzed by these HPs when they are activated by peroxides. Hence, H_2_O_2_ is generally chosen as a target compound to assess the bioactivity of these HPs. The immobilized HPs exhibited electro-catalytic functions related to the decline of H_2_O_2_ when the mediator hydroquinone is present. The response of the developed biosensor to H_2_O_2_ was ascribed to the quantity of Au nanoparticles in the nanocomposites [201].

In another study, Eisele et al. [202] applied BC as external lamella in glucose biosensors for the amperometric determination of products of glucose oxidase reactions. A prolonged stability (200 h) was obtained with diluted blood (1:10), which was longer than that of the commercial Cuprophan^®^ substrate (stability 30 h) with the same condition. Similar results in terms of stability were obtained for undiluted human blood. The stability of the BC-based biosensor was more than 24 h, which was longer compared to Cuprophan^®^ (3–4 h). They found that modifying BC membrane with polyamide extended the measuring range of the biosensor for glucose up to 170 mM [202].

### 4.5. Other Biomedical Applications of BC-Based Materials

Apart from the biomedical applications explained above, BC and its composites have also been utilized in several other biomedical applications, including cancer targeting [203], cornea substitute [198], biological diagnosis [204] and biology–device interfaces [205]. In a study [206], nanogel complexes have been prepared using poly-*N*-isopropyl acrylamide-co-butyl methacrylate (PNB) and various concentrations of BC by a surfactant-free emulsion polymerization method. These nanogel biomaterials exhibited reversible thermosensitive phase behaviors, which changes from an expanded gel to a condensed gel with an increase in temperature. Strong hydrogen bonds between the nanogels and water molecules appeared at lower temperatures, which resulted in completely expanded nanogels. The amount and strength of hydrogen bonding interactions decreased with an increase in temperature. This led to the creation of opaque condensed nanogels. These nanogel complexes can be an ideal biomaterial for an extensive range of medical applications, including injectable biomaterials and vascular embolization interventional therapies [206]. 

Shi et al. [205] introduced an electroactive hydrogel, which is a combination of conductive polymers and BC to make a bio-device interface. In this research, polypyrrole or polyaniline were polymerized electrochemically on the BC hydrogel surface. This produced a biphasic Janus hydrogel with voltage-responsive characteristics. These types of hydrogels, which respond to voltage changes, can provide a base for combining microelectronics with biology to make implantable devices for upcoming recreational medicine. In a more recent study, BC has also been utilized as a biological membrane system for recombinant *E. coli* strains for probable applications in chemical and biological diagnosis [204].

## 5. Conclusions

Bacterial cellulose is an attractive candidate for biomedical applications due to its unique specific structure and properties, along with its biocompatibility. Due to its easy fermentation procedure, large-scale BC production appears to be possible, although the specific engineering details still need to be explained. Many studies have been conducted on creating a mass synthesis process for BC, which significantly reduces the production cost. Large-scale industrial production of BC is still not feasible. Similarly, more biochemical and genetic studies need to be accompanied in order to fully comprehend and enhance the cellulose production process. 

After reviewing the literature, it was obvious that BC and its composites could be applied for tissue engineering scaffolds, transdermal applications, diagnostic biosensors, and drug delivery systems. This provides evidence that BC could be used in more areas than previously thought, with a multidisciplinary approach needed to fully exploit the medical potentials of BC.

## Figures and Tables

**Table 1:** BC/nanomaterial composites and their applications.

BC	NPs	Methods/Materials	Application	Ref.
*G. xylinus* strain 1.1812	Ag	BC/Ag NPs composite prepared by hydrothermal synthesis in a gentle heating system	Antibacterial	[110]
*A. xylinum*	Ag	Using of sodium borohydride for reduction of Ag^+^	Wound healing with increased antimicrobial activity	[111]
*G. xylinus*	Ag	Using of borohydride for reduction of Ag^+^	Antimicrobial activity.	[112]
*A. xylinum*	Ag	Using of sodium citrate for reduction of silver nitrate	Surface enhanced Raman scattering (SERS)	[113]
*A. xylinum*	Ni	-	Magnetic	[114]
*A. xylinum*	Au	-	Security paper	[115]
BC	Au	Using poly(ethyleneimine) (PEI) as the reducing and linking agent	Biosensors (bioelectroanalysis and bioelectrocatalysis)	[116]
*G. xylinus* 700178	Fe_3_O_4_	Polyethylene glycol (PEG) was used to cover the FeNPs	Blood vessels	[117]
*A. xylinum*	Fe_3_O_4_	-	Material for immune selective absorption	[118]
*G. xylinus*	Fe_3_O_4_	Using of Na_2_SO_3_ as a reducing agent	Electromagnetic absorbing materials	[119]
*A. xylinum*	Fe_3_O_4_	PEG was used to cover Fe_3_O_4_ NPs	Electronic actuators, Information storage, Electromagnetic shielding coating	[120]
BC	CO	-	Flexible power source	[121]
*A. xylinum* TISTR No. 975	ZnO	NH_4_OH was used as a reducing agent	Anti-bacterial activity, Water disinfection	[122]
*A. xylinum*	ZnO	-	Photocatalysis	[123]
*G. xylinum*	V_2_O_5_	Nanoparticles were prepared by a sol-gel method	Biosensors and optical devices	[124]
*xylinum* NUST5.2	TiO_2_	-	Photocatalytic activity	[125]
*G. xylinum*	TiO_2_	-	Purification of drinking water, air cleaning	[126]
BC	Cu	-	Antimicrobial activity	[127]
*G. xylinum*	SnO_2_	-	Electrochemical lithium ion batteries	[128]
*G. xylinum*	CdS	-	photocatalyst, novel luminescence and photoelectron transfer devices	[129]
*G. xylinum*	CdSe	-	Photoluminescence	[130]
*A. xylinum* NUST4.2	Pd-Cu	Potassium borohydride was used as a reducing agent	Catalytic activity	[131]
*G. xylinum*	Pd	Laccase (Lac) and Nafion added into Pd/BC	Biosensor	[132]
*G. hansenii* ATCC 10821	Pd	Sodium dithionite incubated with Pd/BC	Catalyze, fuel cell	[99]
*A. xylinum* NUST4.2	Pt	Liquid phase chemical deoxidization method	Fuel cell, biosensors	[133]
